# High gastrointestinal microbial diversity and clinical outcome in graft-versus-host disease patients

**DOI:** 10.1038/s41409-018-0254-x

**Published:** 2018-06-14

**Authors:** Florent Malard, Cyrielle Gasc, Emilie Plantamura, Joël Doré

**Affiliations:** 1 Service d’Hématologie Clinique et de Thérapie Cellulaire, Hôpital Saint Antoine, APHP, Paris, 75012 France; 2 MaaT Pharma, Lyon, 69007 France; 3 MetaGenoPolis, INRA, Université Paris-Saclay, 78350 Jouy en Josas, France

## Gut microbiota diversity: the cornerstone of human health

**Fig. 1:**
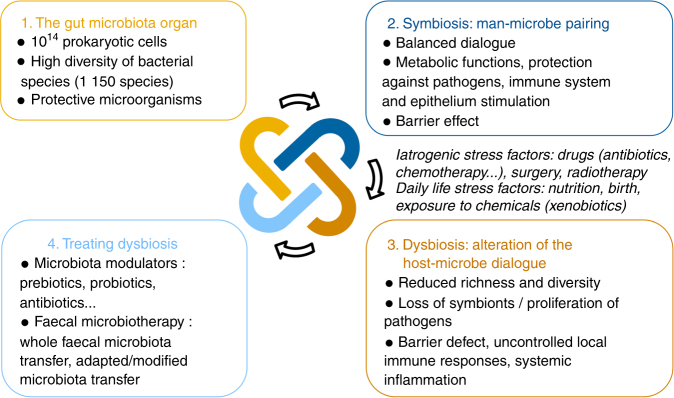
The gut microbiota: from massive bacterial diversity to focused therapeutic target

The human gastrointestinal tract is colonized by approximately 100 trillion prokaryotic cells, most of them being obligate anaerobic bacteria (Fig. 1). Among these bacteria, three main phyla, the Bacteroidetes, Firmicutes, and Actinobacteria, totalize more than 90% of the community and dominate the gut microbiota of healthy subjects. However, the composition of this microbiota is highly diverse and variable at low taxonomical levels (genus and species) between individuals. Indeed, studies based on next-generation sequencing (NGS) approaches demonstrated that the gut microbiota harbors between 1000 and 1150 different bacterial species at the population level, with each individual carrying at least 160 species [1].

This microbial diversity results from the co-evolution between microbial communities and their hosts. In healthy subjects, this close symbiotic relationship is highly beneficial for both parties (Fig. 1) [2]. Indeed, gut microbiota exerts essential functions in digestive metabolism, protection from pathogen colonization, immune system stimulation, and trophic functions at epithelium level. Thus, the balance of commensal microorganisms in the gut microbial ecosystem is essential for the maintenance of host-microbiota homeostasis and barrier effect against pathogens.

Disruption of this balance, called dysbiosis, can alter this mutualistic relationship and promote pathological conditions involving uncontrolled local immune responses and potentially systemic inflammation (Fig. 1) [3]. Dysbiosis often arises from iatrogenic factors such as surgery or oncology-associated treatments, and particularly drugs, including chemotherapy and broad-spectrum antibiotics, which dramatically alter the structure of the microbial ecosystem [4]. This shift in gut microbiota composition is characterized by a reduction of overall microbial diversity, a disruption of beneficial bacteria that support host defenses (e.g., Firmicutes), and a rise in dominance of bacterial species usually subdominant, including some pathogens and pathobionts (e.g., *Clostridium difficile*, some Enterobacteriaceae) and multidrug-resistant (MDR) bacteria.

We herein highlight the evidence that microbial diversity is the cornerstone of a good health, while a decreased diversity is often related to poor clinical outcomes, and especially in graft-versus-host disease (GvHD) patients.

## Higher gut microbial diversity is strongly associated with increased survival in GvHD patients

Allogenic hematopoietic stem cell transplant (allo-HSCT) is an effective treatment for hematopoietic malignancies and inherited hematopoietic disorders, and is considered to be the most effective tumor immunotherapy available to date [5]. However, T lymphocytes derived from transplanted stem cells can attack tissues of the recipient host resulting in GvHD, one of the major complications of allo-HSCT associated with significant mortality (15–25% of deaths after allo-HSCT). Patients undergoing allo-HSCT can be simultaneously exposed to cytotoxic chemotherapy, total-body irradiation, immunosuppressors, and broad-spectrum antibiotics that can cause dramatic alterations of the intestinal microbiota and varying degrees of damage to the intestinal mucosa, leading to breaches in host defences [4, 6–8].

**Fig. 2:**
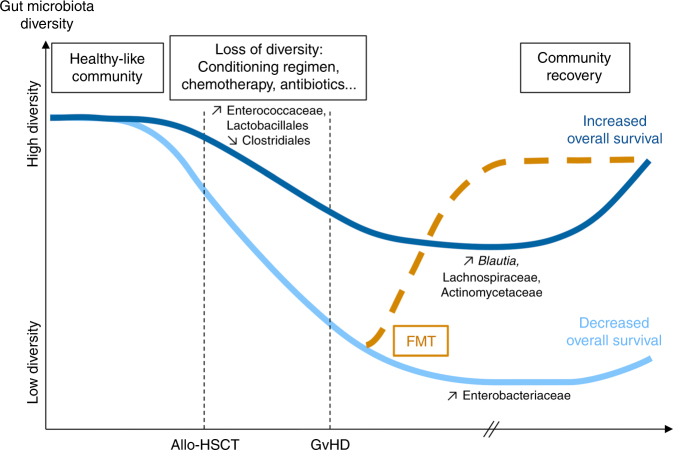
Disruption of the gut microbiota over the course of allo-HSCT

Examination of the intestinal microbiota of patients before allo-HSCT, using NGS, demonstrated that the gut microbiota composition of recipients correlates with the approximate microbiota profiles of healthy individuals in terms of species richness and diversity [9–11] (Fig. 2). Over the course of allo-HSCT, patients show profound shifts in microbial communities marked by a significant reduction in overall microbial diversity, with an approximate decrease of 30% of species richness [11–13]. Indeed, a significant shift toward Enterococcaceae is usually observed in patients after allo-HSCT, and interestingly, this shift is particularly prominent in patients that subsequently develop GvHD. Similarly, increases in Lactobacillales and decreases in Clostridiales also happen after allo-HSCT for GvHD subjects [10, 12, 14].

This loss of microbial diversity often results in a reduced range of microorganisms in dominance, or even a single bacterial species that supplants the previously rich and complex consortium of microorganisms. As a result, bacteria such as vancomycin-resistant *Enterococcus*, viridans group *Streptococcus*, and various Proteobacteria, commonly encountered dominating, can enter the bloodstream and cause septicemia, particularly in times of neutropenia and gut mucosal barrier injury [9, 15]. Moreover, a study conducted on pediatric patients demonstrated that the gut microbiota of GvHD patients before allo-HSCT shows lower species diversity and richness than non-GvHD patients, with specific gut microbiota signatures and notably a reduced abundance of members of Bacteroidetes phylum and a loss in the abundance of health-associated bacteria such as *Faecalibacterium* and *Ruminococcus* [13]. Thus, these results demonstrate that the diversity of the gut microbiota of patients correlates with the occurrence of medical complications resulting from allo-HSCT, especially the risk of infection and GvHD.

The diversity of the gut microbiota also plays a key role in overall survival after allo-HSCT, and in GvHD patient outcome. Indeed, despite the medical treatment administered, approximately 30% of patients undergoing allo-HSCT maintain a high microbial diversity throughout the course of transplantation. A major study conducted on 80 allo-HSCT patients showed that a high diversity of the intestinal microbiota at the time of engraftment is associated with an increase of overall survival and a reduced non-relapse mortality, independent of known predictors such as pre-transplant comorbidity and disease status [15]. Indeed, the overall survival at 3 years was 67% for patients with a high microbial diversity at the time of engraftment, 60% for patients with an intermediate diversity, and 36% for patients with a low microbial diversity. Similarly, another study demonstrated that a low microbial diversity after allo-HSCT is associated with an increased non-relapse mortality and a reduction of overall survival [14]. The importance of a high microbial diversity is confirmed by studies on GvHD patients for whom an increased microbial diversity between allo-HSCT and development of GvHD is associated with a reduced GvHD-related mortality [16]. These studies also evidenced that in addition to diversity, composition of the gut microbiota of patients who remain alive after allo-HSCT significantly differs from the microbiota of patients who do not survive. Indeed, increased amounts of bacteria belonging to the genus *Blautia* and to the Lachnospiraceae and Actinomycetaceae families are observed in patients who survived during the follow-up period, whereas greater abundance of Gammaproteobacteria, including Enterobacteriaceae is observed in deceased patients [15, 16]. Thereby, diversity and composition of the gut microbial community clearly appear to harbor reliable predictors of the overall survival after allo-HSCT and in GvHD patients (Fig. 2).

Confirming these results, administration of high loads of broad-spectrum antibiotics, which dramatically modify the bacterial diversity, was shown to be negatively correlated with overall survival of patients [17, 18]. Indeed, a cumulative exposure to antibiotics has been associated with an increased GvHD-related mortality at 5 years when using antibiotics effective against anaerobic bacteria such as piperacillin and tazobactam (19.8% mortality for treated patients versus 11.9% for untreated patients). Piperacillin and tazobactam, which are highly active against obligate anaerobic bacteria, accentuate the perturbation of the gut microbiota composition, with greater loss of several major bacterial populations such as Bacteroidetes and Lactobacillales. On the contrary, antibiotics like cefepime and aztreonam, with a reduced activity against anaerobes and a lower impact on microbial communities, are significantly correlated with reduced GvHD-related mortality [17]. Thus, this study indirectly points out a correlation between GvHD-related mortality and reduced microbiota diversity. Similarly, a study conducted on 621 patients who underwent allo-HSCT demonstrated that an antibiotic treatment before allo-HSCT was associated with a higher transplant-related mortality (34%) compared with an antibiotic treatment post allo-HSCT (21%) or no antibiotherapy (7%) [19]. These results suggest that differences in the spectrum of activity of antibiotics used and the timing of antibiotic treatment might modulate the severity of GvHD and its outcome through modifications of the gut microbiota, highlighting once again the role of microbial diversity in clinical outcome of these patients.

## Fecal microbiota transfer: toward health improvement through the restoration of a diverse gut microbiota

As illustrated with allo-HSCT treatment and GvHD complication studies, given the importance of the intestinal microbiota, solutions to maintain a high microbial diversity could lead to significantly improved clinical outcomes in many diseases. First, it can be hypothesized that limiting administration of broad-spectrum antibiotics could reduce the dramatic disruptions of the gut microbiota composition, consequently diminish the severity of many diseases including GvHD, and thereby reduce mortality. Then, modulating the gut microbiota to restore the diverse commensal microbial populations lost during disease treatment could offer novel therapeutic possibilities.

The establishment of bacteriotherapies such as fecal microbiota transfer (FMT), consisting in administering fecal material from a healthy donor to a patient with an altered gut microbiota, could be an efficient tool for dysbiosis correction. The purpose of FMT is to increase microbial diversity and restore a healthy microbiota [1], to re-establish a symbiotic dialog between intestinal microbiota and the host, and consequently to improve clinical outcomes and overall health (Fig. 1). For instance, treating dysbiotic GvHD patients with FMT using a fecal sample associated with a highly diverse microbial community could be a relevant strategy to restore a healthy gut microbiota and improve their health (Fig. 2). Thus, in case of allogeneic FMT, the selection of donors could therefore be guided by the richness and diversity of their intestinal microbiota in order to maximize the expected benefits of FMT for the patient. Moreover, this strategy could promote the reintroduction in the gut ecosystem of health-promoting microorganisms that might reduce the severity of GvHD and the risk of death, such as *Blautia* or the Lachnospiraceae and Actinomycetaceae [15, 16]. This could be achieved by conventional FMT but also by FMT using fecal material enriched with specific health-promoting microorganisms.

These potential beneficial effects of FMT are supported by two recent small case series, which illustrated that FMT is effective in GvHD patients, with an improvement of gastrointestinal symptoms and a reduction or disappearance of diarrhea, associated with a reconstruction of the gut microbiota [20, 21]. Another recent report confirmed and extended the 7 cases reported so far with 5 complete responses and 3 partial responses among 11 treated patients, with excellent safety profiles [22]. Finally, with the restoration of a diverse microbiota, FMT could limit pathobiont domination and consequently enhance resistance to infection by intestinal pathogens through a restored barrier effect of the gut microbiota. FMT is currently used for treatment of infections with *C. difficile* or for MDR bacteria decolonization, and has started to demonstrate its safety and efficiency on allo-HSCT patients [23–26]. Alternative sterile FMT approaches, which consist in using filtered stool supernatants free of any microorganism, could also be very effective and safe for the treatment of immunocompromised GvHD patients [27].

Thus, growing evidence suggests that through the restoration of a rich and diverse gut microbiota, FMT could be a promising treatment for many digestive or extra-digestive pathologies involving the gut microbiota symbiosis. As it could be used for the treatment of GvHD as illustrated here, FMT could also be of interest for the treatment of patients undergoing cancer therapy for whom the diversity of the gut microbiota significantly influences the efficiency of immunotherapies [28–30], in patients suffering from severe alcoholic hepatitis for whom the gut microbiota diversity is related to clinical outcome [31], or even for patients admitted in intensive care units for whom a diverse gut microbiota could prevent bacterial infections and sepsis [32, 33].

Furthermore, new therapeutic strategies combining bacteriotherapies such as FMT that modulates the gut microbiota, and conventional drugs targeting the host physiology and immune system, offer promising perspectives for effective patient care through the bilateral restoration of the host-microbiota homeostasis.
